# The BCG *in situ* study – novel techniques applied to a 100-year-old vaccine

**DOI:** 10.1016/j.mex.2025.103712

**Published:** 2025-11-07

**Authors:** Frederik Schaltz-Buchholzer, Ole Bæk, Amrit Singh, Elsi Cá, Isaquel da Silva, Peter Aaby, Christine Stabell Benn, Tobias R. Kollmann, Nelly Amenyogbe

**Affiliations:** a Bandim Health Project, Apartado 861, 1004 Bissau codex, Bissau, Guinea-Bissau; b Bandim Health Project, OPEN, Department of Clinical Research, University of Southern Denmark, Campusvej 55, 5230 Odense M, Denmark; c Comparative Pediatrics, Faculty of Health Sciences, University of Copenhagen, Grønnegårdsvej 15, 1870 Frederiksberg C, Copenhagen, Denmark; d BRIDGE Translational Excellence Programme, Faculty of Health Sciences, University of Copenhagen, Denmark. Dyrlægevej 68, 1870 Frederiksberg C, Denmark; e Department of Anesthesiology, Pharmacology and Therapeutics, University of British Columbia, Medical Sciences Block C, 217 - 2176 Health Sciences Mall, Vancouver BC V6T 1Z3, Canada; f Danish Institute for Advanced Study, University of Southern Denmark, Fioniavej 34, 5230 Odense, Denmark; g Dept. of Microbiology & Immunology, Dalhousie University, Halifax B3H 4R2, Canada; h Canadian Centre for Vaccinology, IWK Health Centre, Halifax, Canada; i Breastfeeding & Immunology, The Kids Research Institute, Perth, Australia

**Keywords:** Bacillus Calmette-Guérin, Non-specific effects, Punch skin biopsy, Liquid biopsy, Localized cellular and molecular skin effects, Multiomics

## Abstract

The use of Bacillus Calmette-Guérin (BCG) vaccine against tuberculosis (TB) spans more than a century. Besides protection against severe paediatric TB, randomized trials and novel advances within innate immunology documented that BCG has beneficial non-specific effects, providing protection against non-TB infections. Since paediatric intradermal BCG vaccination has proved unable to contain adult pulmonary tuberculosis, several novel TB vaccines are under development, most of which build upon BCG.

BCG’s status as an essential remedy against TB will therefore be maintained, but despite many decades of near-universal intradermal use, the local responses to BCG in the skin have not been thoroughly elucidated.

We therefore developed appropriate methods to capture the localised skin events at the cellular and molecular level after intradermal BCG vaccination. This work informs future studies to identify the immunological events induced following administration of BCG to accelerate development of improved or new vaccines against *Mycobacterium tuberculosis (Mtb)*. We employed advanced technologies such as spatial transcriptomics on skin tissue punch biopsies and cell-free plasma transcriptomics (liquid biopsies) to characterize both the local (*in situ*) skin and systemic (peripheral blood) response to BCG via:

• Whole blood transcriptomics and epigenetic analysis
• Blood immune cell characterization
• Plasma proteomic and metabolomic analysis

## Graphical abstract

As requested, the graphical abstract is uploaded separately.

**Figure:**
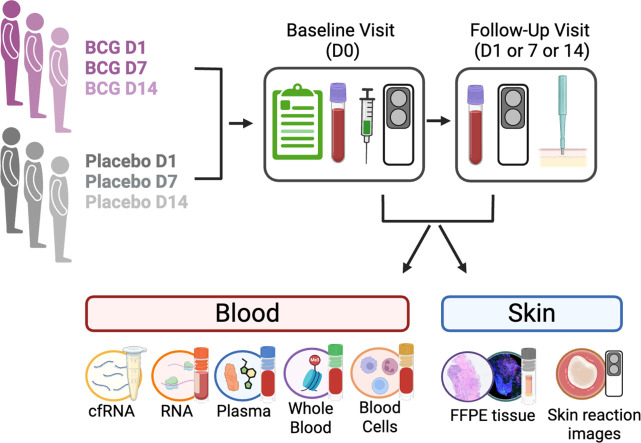

**Specifications table**

**Table:** 

**Subject area**	Immunology and Microbiology
**More specific subject area**	Examination of immunological events at the cellular and molecular level following BCG vaccination
**Name of your protocol**	BCG *in situ*: Testing if BCG vaccination induces changes in the skin and in blood that predict scar formation and with that health benefit.
**Reagents/tools**	BCG (Denmark strain, AJ vaccines, Denmark) PAXGene blood RNA preservative DNase/RNase DNA Lo-Bind tubes DNase/RNase-free wide-bore pipette tips Smart Tube system multiomics Fixable Viability Dye sterile dPBS RNAase inhibitor (RNAlater, ThermoFisher) qPCR Data Analysis Suite, GeoMx platform R Statistical Computing Environment (version 4.4.1) Python (version 3.12) Dermatoscopic imaging (Google Pixel Phone + dermatoscopic device)
**Experimental design**	Randomized experimental study to capture the localized skin events and systemic (peripheral blood) response to intradermal BCG vaccination
**Trial registration**	Not applicable.
**Ethics**	The study protocol was approved by the National Committee for Ethics in Health Research of Guinea-Bissau (CNES), reference number 027/CNES/INASA/2023. All participants provided written informed consent prior to participation and the study was conducted in accordance with the Declaration of Helsinki.
**Value of the Protocol**	• The study protocol addresses the knowledge gap around the initial local events both at the site of BCG vaccination and systemically. • The knowledge created by the methods outlined in this protocol enables future large-scale application in clinical studies. • In combination, the data generated may help identify the immunological mechanisms responsible for BCG-mediated protection against both TB and other infections, informing strategies to improve or even develop novel vaccines against *Mtb* and leveraging BCG’s non-specific effects against other infections.

## Background

Infection with *Mycobacterium tuberculosis* (*Mtb*) is a leading cause of infectious death in the world, with >10 million new cases of tuberculosis (TB) and 1.25 million deaths in 2023 [1]. The Bacillus Calmette–Guérin (BCG) vaccine was developed over 100 years ago and is the only licensed vaccine for TB control, administered to >120 million people every year [2]. Developed by attenuation of *Mycobacterium bovis*, BCG vaccination leads to the formation of adaptive immunity that recognizes mycobacterial antigens including *Mycobacterium lepra*; but it remains unclear how adaptive immunity is first initiated and then contributes to protection from initial *Mtb* infection and/or subsequent development of TB disease [3]. BCG administered early in life is protective against severe paediatric TB such as meningeal and miliary TB, but protection against adult pulmonary TB is highly variable, indicating that age is an important variable [4]. BCG has also been shown to provide protection from non-mycobacterial infections via *non-specific* (also occasionally referred to as *pathogen-agnostic* or *heterologous)* effects, indicating that more than mycobacteria-specific immunity is achieved following BCG administration [5]. BCG thus persistently alters gene expression in human hematopoietic stem cells, pushing their differentiation towards the myeloid lineage also beyond 90 days following immunisation [6]. Beneficial non-specific effects has been demonstrated for the *BCG-Danish* strain in trials from Guinea-Bissau [7,8], Uganda [9] and India [10]. Contrary to the effects of BCG-Danish, two Indian trials of at birth BCG-Russia vs delayed vaccination reported no beneficial non-specific effects [11].

BCG was initially administered orally to human newborns in multiple doses, but the intradermal route of administration became the preferred delivery route during the last century, formally implemented by WHO from 2004 [[12], [13], [14]]. While the TB-specific protection following intradermal BCG vaccination appears unrelated to the formation of a BCG scar in the skin [15], the non-specific health benefits of BCG correlate with the recipient developing a BCG scar, and the size of that scar [16,17]. However, despite BCG having been in use for over a century, the local responses in the skin, i.e. at the site of intradermal BCG administration, have not been thoroughly assessed beyond a handful of studies. The few existing studies are limited to histological examination of hematoxylin-eosin stained skin biopsies, quantitative PCR and bacterial cultures to determine the BCG load at the injection site, with BCG clearance from the skin having been proposed as a human challenge model that can provide a correlate of protection against *Mtb* disease [[18], [19], [20], [21], [22]]. Despite the documented importance of the early events in the skin for all-cause survival, it remains unknown how the immunological cascade induced by BCG injection in the skin leads to protection from pathogen-specific (i.e., *Mtb*) and non-TB infections. Lastly, in adult animal models, BCG provides better protection against *Mtb* infection and TB disease when provided intravenously, when compared to intradermal or subcutaneous administration, indicating an importance of the administration route, and with that events at the site of administration, as a crucial variable [[23], [24], [25], [26], [27], [28], [29], [30], [31]].

## Description of protocol

### Rationale

The knowledge gap around the initial events at the site of BCG vaccination might have hindered the development of more effective vaccination strategies in the fight against TB as well as other infectious diseases [5,32]. We therefore designed the *BCG In Situ* experimental study to capture the immunological events in the skin following intradermal administration of BCG at the cellular and molecular level. To achieve this, we employed advanced technologies that have only recently become available, such as spatial transcriptomics (voted Nature’s ‘2020 Method of the Year’ [33]) on skin tissue biopsies, as well as ‘liquid biopsies’ to extract the circulating cell-free transcriptome to capture signals from tissue-based processes in peripheral blood [[34], [35], [36], [37]]. To our knowledge, these approaches have never been applied to vaccines, and specifically not to BCG. These two cutting edge technologies were coupled with assessment of the systemic response to BCG via whole blood transcriptomics, blood immune cell characterization, whole blood epigenetic analysis, as well as plasma proteomic and metabolomic analysis, as described previously [[38], [39], [40]]. Our hypothesis was that intradermal BCG induces molecular and cellular changes in the skin and that these local reactions are reflected in changes that are detectable in peripheral blood samples. For this initial study, the parallel approach of simultaneously capturing local and systemic events specifically aimed to identify readily accessible biomarkers in peripheral blood that reflect local tissue events. This enables future large-scale application in subsequent clinical studies to relate the identified biomarkers of local events to clinically relevant endpoints. Furthermore, our approach informs future work that can possibly identify the immunological mechanisms responsible for BCG-mediated protection against TB and other infections, informing strategies to improve on or even develop novel vaccines against Mtb and other infections.

## Methods/design

### Study overview

The BCG In Situ study was a proof-of-principle, individually randomised experimental study enrolling 42 adult women in Guinea-Bissau that are HIV-negative, non-pregnant, and without signs of active TB. The choice of only recruiting adult women in this pilot was to reduce sex-based variability; in future studies, both sexes and children should be included. Guinea-Bissau was chosen as the study site because of extensive previous experience with BCG studies at the Bandim Health Project, and because Guinea-Bissau is a TB-endemic country where it was known to be feasible to recruit participants both with and without BCG scars. We assessed the local skin response to BCG, as well as changes that occur in peripheral blood. Of the 42 participants, 30 received BCG at the time of the inclusion and the remaining 12 received a saline placebo. All participants in the placebo group were offered BCG at the end of the trial.

#### Intervention

Participants received BCG (Denmark strain, AJ Vaccines, Denmark) or standard sterile saline as placebo control, administered intradermally in the right deltoid region.

### Primary and secondary objectives

**Primary Objective**

- To assess the molecular changes induced by BCG in the skin at day 1, 7, and 14 following vaccination. This include comparing skin responses and molecular changes over time between placebo and BCG-vaccinated groups.

**Secondary Objectives**

1. To apply an unbiased systems biology (multiomics) approach to identify peripheral biomarkers that are associated with the local skin response.
2. Assess the feasibility of performing a skin biopsy collection protocol and deep molecular phenotyping on multiple tissues collected in Guinea-Bissau, a low-resource setting in Sub-Saharan Africa.

### Setting

The Bandim Health Project’s (BHP, www.bandim.org) Health and Demographic Surveillance System (HDSS) site continuously surveys a population of ∼100,000 individuals in six suburbs in Bissau, Guinea-Bissau, with home visits and surveillance at HDSS health centers and the nearby national hospital. The BHP has a long track record of conducting large-scale vaccine RCTs with overall and infectious disease morbidity and mortality as outcomes [8,[41], [42], [43]].

### Recruitment

A list of potentially eligible individuals was first drawn from the BHP HDSS database (female, non-pregnant, ≥18 years of age). These were visited at home, where a brief explanation of the study is provided. Participants who were not acutely ill and who were willing to participate were asked to come to the BHP Center for enrolment. Here, they were provided with a written explanation of the study in Portuguese as well as a full verbal explanation in Portuguese Creole by a Bissau-Guinean study physician. In case the person was not fluent in Portuguese Creole, an interpreter assisted. It was explained that BHP is conducting an experimental study to investigate whether BCG skin reaction formation, and with that potential health benefits from BCG, can be predicted shortly after vaccination. Participants were furthermore informed about the randomization procedure, the follow-up procedures, and the sample collection procedures (blood and skin). It was explained that after numbing the skin area with ice, a small 2 mm biopsy of the skin would be collected via a standard biopsy, a procedure that does not cause more discomfort than that associated with the skin prick for administration of an anaesthetic [[38], [39], [40]]. The associated risks such as bleeding or infection of the site are similar to an intravenous blood draw. It was also explained that in addition to the skin biopsy, we asked participants to donate a small amount of blood (4 ml) on the day of inclusion and at one follow-up session. An introductory video with explanation of the intradermal injection and biopsy sampling procedures was prepared in Portuguese and shown to the participants during the informed consent process. Participants were further informed that they would not receive any compensation for their participation in the study.

Provided informed consent, HIV-negative non-pregnant women were interviewed regarding their medical history and had their BCG scar status (scar present/absent) visually inspected (deltoid region of both arms, according to the vaccination policy). The presence of a scar was interpreted as indicative of previous successful BCG vaccination. We collected phone numbers of the participant and 1-3 relatives, so that participants could be reached for follow-up. BHP staff ensured that half of the 42 participants had a BCG scar, and half did not, since the presence of a BCG scar could modulate the In Situ responses to BCG.

### Baseline and follow-up visits

At the BHP Centre, the team consisting of medical doctors and nurses was present to conduct a clinical examination and study procedures.

#### Screening for HIV

Was performed by blood HIV rapid antigen testing with the screening result provided to the participant by an attending physician who was available for immediate consultation in case the HIV test was positive. Such cases were to be referred for further testing and consultation at the national HIV clinic, which provide medical care according to the national standard of care.

#### Screening for TB

Adult TB causes coughing, most often a productive cough, haemoptysis, weight loss, night sweats, chest pain, fever, and tachycardia. At physical examination, one can find a lower mid-upper-arm circumference, a low body mass index (BMI), fever, and pathological lung auscultation. We screened eligible participants for active Mtb infection by means of a clinical interview, examination, and scoring using the previously validated Bandim TB score [44], which is based on the patient’s history and the physical examination. If there is clinical suspicion of active TB (Bandim TB score >2), the individual was excluded from participation in the study due to the theoretical risk of an adverse reaction to BCG, and instead referred to the main TB clinic in Bissau to receive appropriate medical care, in accordance with national guidelines. It was not possible for our team to perform Interferon-gamma release assays such as T-spot to rule out latent TB in the participants.

#### Pregnancy test

Pregnancy was assessed by a rapid human choriogonadotropin urine test. Any women with positive tests were referred to the antenatal programme at their local health centre.

#### Inclusion criteria

Adult women ≥18 years of age who provided informed consent and did not conform to any of the exclusion criteria were considered eligible for inclusion. Experienced BHP staff ensured, through visual inspection of both upper arms, that half of the 42 participants had a BCG scar and half did not.

#### Exclusion criteria

Women who were mild to severely ill and/or had been in contact with the health system (hospital or health centre) during the 2 weeks prior to the enrolment interview were excluded. Likewise, as mentioned above, those that showed signs of TB, were HIV positive or pregnant, were excluded. Women who had received a live vaccine within the past month were asked to return when one month had passed before participating.

### Group allocation

**Fig. 1:**
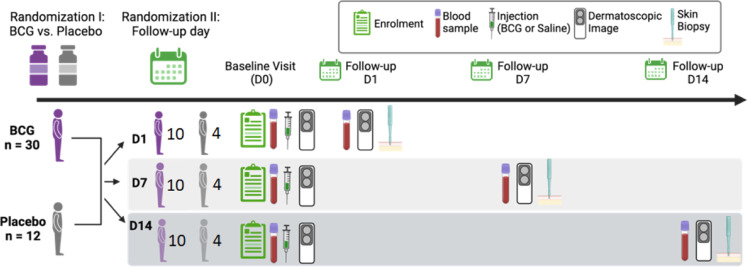
**BCG In Situ Study Overview.** Note: half of the BCG and half of the Placebo groups were BCG scar positive based on visual inspection at enrolment.

Those who wished to participate in the study were asked to sign the consent forms (written signature or fingerprint). Eligible participants drew lots randomly allocating them to receive either BCG (N=30, 15 with and 15 without a BCG scar) or placebo (N=12, 6 with and 6 without a BCG scar). Further, participants were randomly allocated to return for the skin biopsy and blood draw at either 1-, 7- or 14-days post-inclusion (Fig. 1).

Participants received a BHP health card with a unique number, to be brought with them at follow-up for secure identification. This card gave access to basic medications and consultations in case the participant should fall ill during a period of 12 months following enrolment. The 12 placebo recipients were informed that they could receive BCG at the end of the study.

#### Intervention

Participants received BCG (standard 0.1 mL adult dose) or 0.1 mL sterile saline as placebo control. Following intradermal administration, the height and width of the injection site was measured with a ruler to document the size of the inoculum, followed by dermatoscopic imaging of the injection site.

#### Follow-up

At the follow-up visit, another dermatoscopic image of the skin area prior to biopsy was taken, followed by collection of skin and blood samples (Fig. 1). At enrolment and at follow-up, data regarding physical health, medication use, consultation and hospitalization were collected. Furthermore, at each eventual consultation to the special clinic between visits caused by disease, a form would be filled out with information about the consultation, including the symptoms/complaints, the diagnosis and the recommended treatment. The participants were also be told that in case of illness, they should come to the BHP Center, where the clinic is staffed with a medical doctor. In case of acute severe illness during non-clinic hours, a telephone number was provided for a medical doctor affiliated with BHP. At the clinic, participants were provided free medical consultations and essential medications for 12 months. In case of serious illness, BHP would assist in organizing transport and admission to the main hospital.

### Biospecimen collection, processing and storage

#### Blood draw

Each participant donates blood twice over the course of the BCG In Situ study: at baseline and at one follow-up visit, with 10 BCG and four placebo participants per time point (Fig. 1). Both blood draw and skin biopsy collection took place in a health centre. Blood samples were drawn into 4.0 mL K2 EDTA vacutainers (BD 367839).

#### Blood processing

**Fig. 2:**
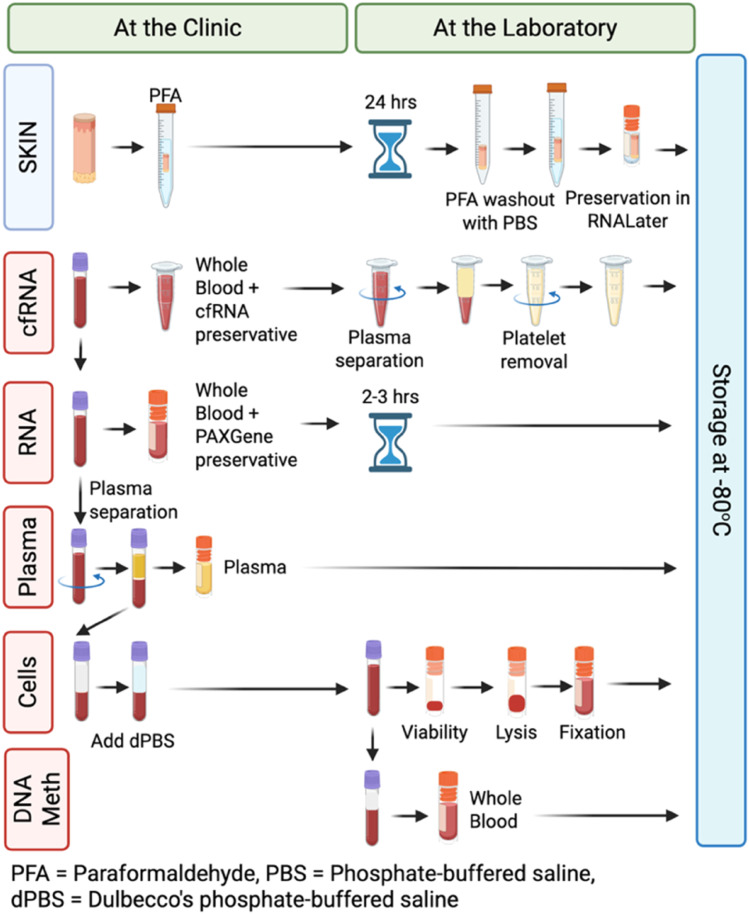
Overview of BCG *In Situ* sample processing procedures first at the clinic and then at the laboratory, followed by storage at -80°C.

The blood processing protocol was performed in two stages. First, at the site of blood sample collection (the health center), whole blood was preserved for RNA and cfRNA analysis, and then plasma was separated and aliquoted. The biosamples were then transported to the BHP laboratory and -80°C sample storage site, where the remainder of the cfRNA processing, cellular preservation, and whole blood aliquoting was performed (Fig. 2). RNA was first preserved by placing 500 µL whole blood into 1380 µL PAXGene blood RNA preservative (Norgen 63950). After at least 2 hours at room temperature, blood PAXGene samples were placed at -80°C. To preserve cfRNA, 1.3 mL blood was placed into 247 µL cfRNA preservative (Norgen Cat. 63950) using DNase/RNase-free DNA Lo-Bind tubes (Sarstedt 72.694.700) and mixed gently using DNase/RNase-free wide-bore pipette tips (Axygen T-1005-WB-C-R-S). This step minimized cfRNA degradation between sample collection and sample arrival at the processing laboratory 3-6 hours after blood draw.

At the laboratory, the cfRNA preservative-blood mixture was centrifuged at 460 x g for 20 minutes and the top 90% of the plasma lifted to a new 1.5 mL tube (Eppendorf 22431021). This tube was spun again at 2600 x g for 15 minutes to yield cell free plasma. Taking care to avoid the pellet, the plasma was transferred to a new 1.5 mL microtube (Eppendorf 22431021) and stored at -80°C. After the RNA and cfRNA aliquots had been separated (at the clinic), the remaining blood was centrifuged at 1000 x g for 10 min, and plasma removed in two equal aliquots of up to 500 µL each. Once the plasma had been removed, an equivalent volume of phosphate buffered saline (dPBS; Invitrogen PBS74-1-500) was added back to the blood sample to ensure that remaining cells were in their physiological concentration prior to flow cytometry processing. We employed the Smart Tube system to preserve cells for flow cytometry, as previously described [45]. Briefly, a 200 µL blood-dPBS sample was further EDTA-stabilized using 20 µL 20 mM EDTA made by diluting 0.5 M EDTA (Invitrogen 15575020) and Ultrapure water (Invitrogen 10977015), with 2.0 µL Fixable Viability Dye (eBioscience 65-0865-18) added before incubation in the dark for 30 min. Red blood cells were then lysed using 350 uL Stable Lyse buffer (Smart Tube STBLYSE2-250) and immune cells preserved with 1000 uL SmartTube Stable Store buffer (Smart Tube STBLSTORE-1000) before storage at -80°C. From the remaining sample, 500 µL whole blood was then preserved for downstream DNA methylation analysis, also stored at -80°C.

#### Skin biopsy

Each participant donated only one skin punch biopsy over the course of the study, with 10 BCG and four placebo participants per time point (Fig. 1). The previous BCG or placebo administration site was identified by skin reaction formation and/or from photographs taken at the time of administration. The skin was anaesthetised by application of an ice pack for ∼5 minutes and then cleaned with a regular alcohol wipe. The sterile, single-use skin punch (Kai Biomedical SPMKAI697) was then opened and a single, 2 mm biopsy was extracted by staff that were trained in this procedure by a dermatologist who regularly performs the procedure. If required following the biopsy, haemostasis was achieved by applying pressure to the biopsy site. The site was closed by means of sterile wound closure tape (Multigate MTG30353) and then covered with retention tape for 5 to 7 days. The skin samples were processed on site and immediately stored in 4% Paraformaldehyde (PFA) for 24 hours. PFA was prepared by dissolving solid tablets (Sigma 158127-5 G) in sterile PBS (Gibco PBS74-1-500). The samples were then rinsed in sterile PBS (Gibco PBS74-1-500) and frozen in RNAlater (Invitrogen AM7020) for RNA preservation. For analysis, the samples were split and assessed for 1) BCG load by qPCR and 2) histological and spatial analysis of gene and protein expression.

#### Photographic and dermatoscopic imaging

Prior to, and after vaccination at the baseline visit, and also prior to the skin biopsy collection at the follow-up visit, one standard photograph and one dermatoscopic image of the injection site was recorded using a dermatoscope (DermLite DLGL).

#### Sample storage

The processed blood and skin samples were stored in appropriate -80°C freezers before shipment in dry ice for further analysis. During both storage and shipping, samples were kept under temperature-controlled and monitored conditions.

### Data analysis

#### Data management

All data were entered in a secure database. All data exiting Guinea-Bissau were de-identified, including dates. Aside from the analyses described in this research protocol, other exploratory analyses may be conducted, in which case they will be clearly marked as such. All biomaterial from the study is scheduled for destruction after five years.

#### Statistical and bioinformatic analyses

We do not expect data to be normally distributed; consequently, medians with interquartile ranges were calculated and non-parametric tests (Wilcoxon) applied. The integration of the spatial transcriptomics and blood-based multiomics data was analysed using the Data Analysis Suite on the GeoMx platform and the generic R Statistical Computing Environment, as previously described [[38], [39], [40]]. Our analytical strategy is to first apply univariate analysis to identify differentially expressed or abundant features from each data set. The ‘time passed since vaccination’ will be used both as a categorical variable, also to features specific to each time point, and as a continuous variable to identify features that change linearly over time. Data integration will proceed in a structured sequence linking molecular and imaging datasets. For each tissue layer, spatial transcriptomic data will undergo normalization, differential expression testing, and pathway enrichment using curated KEGG (Kyoto Encyclopedia of Genes and Genomes) and cell-marker databases. Plasma cfRNA and dermatoscopic features will be processed in parallel using feature extraction and enrichment analysis. Cross-modal relationships will then be explored with regularized canonical correlation and multiomics factor analysis to identify shared components and temporal trajectories across skin and blood datasets.

#### Sample size

Published literature using spatial transcriptomics and multiomics in interventional randomized clinical studies have shown that participant numbers (n) of ∼5 provides ∼90% power to detect differentially expressed genes between groups at a false discovery rate (FDR) of 5% [46,47]. This very low n (compared to classical clinical trials) relates to the fact that in spatial profiling experiments, the number of cells within a tissue section (for our spatial profiling we measured an average of 500 cells per region of interest) and the sequencing depth are the two key parameters determining statistical power to detect signals of interest [48]. By pooling of the combined 12 placebo recipients (6 with scar, 6 without scar) recruited, we achieve ≥5 participants for each comparison of BCG vs placebo, including for the combinations of follow-up timing and BCG scar status.

#### Skin spatial transcriptomics

The recent development of spatial transcriptomics permits assessment of events at the single-cell level within the 3-dimensional structure of the skin. Applying this novel technology to even a small sample size per time point [with 10 BCG recipients per timepoint of which 5 have a BCG scar and 5 that do not (presumed BCG-naïve), and 4 placebo controls (2 with and 2 without a BCG scar)] permits robust statistical analysis. Each sample is analysed at the level of the single cell; e.g., all skin fibroblasts are counted, and the functional status of each cell determined. Outcomes are thus assessed across thousands of ‘functional units’. This is akin to single cell transcriptomics in blood, which has recently been applied with success in a BCG study featuring with 3 participants per group [49].

#### Dermatoscopic images

Despite the use of BCG for more than a century, there is sparse information available regarding the BCG skin reaction kinetics during the first weeks after vaccination. In addition to the assessment of molecular changes in the skin, we therefore also assessed macroscopic changes at the site of injection after BCG vaccination. All dermatoscopic images were resized prior to feature extraction. We applied pre-trained computer vision models (e.g., ResNet, AlexNet) which extract features reflecting the skin reaction from images.

#### Blood Multiomics

Using available omics datasets, we interrogated all blood compartments for molecules arising from different biological layers. Multiple omics (multiomics) analysis may enable the identification of sets of molecules that are predictive of various outcomes, referred to as signatures. The ability of blood-based assessment of biomarkers for tissue processes has thus been successfully deployed routinely to identify minimal ‘signatures’ that capture complex, tissue-based events [50].

### Analyses for primary objective

**Fig. 3:**
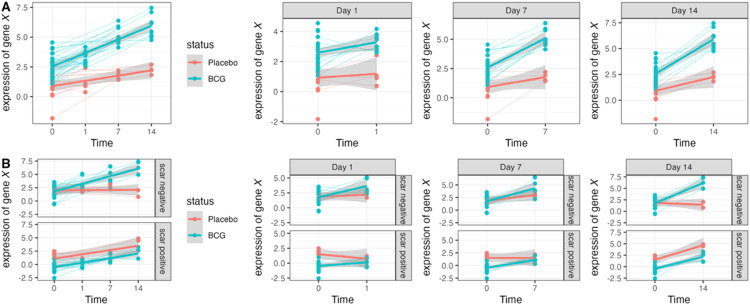
**Hypothetical differential expression analysis for assessing the effects of time, vaccination and scar status on image and blood features using linear mixed-effects model. A.** Representative plots depicting a feature (gene X) with a significant two-way interaction between time and vaccination status. **B.** Representative plots depicting a feature (gene X) with a significant three-way interaction between time, vaccination and scar status. For each model, time were treated as a continuous and categorical variable to allow us to determine changes over time and between each time-point with respect to baseline (day 0).

Given the multifactorial nature of this study, we employed linear mixed-effects models to assess the interactions between time, vaccination and scar status. For each blood omics dataset (e.g., cfRNA, methylation, proteomics) and dermatoscopic data, we first applied a linear mixed-effects model containing time (either continuous or categorical) plus vaccination status as fixed-effects, and participant as the random-effect. A two-way interaction allowed us to identify features that differentially change over time between the placebo and BCG-vaccination groups (Fig. 3**A**). For example, Fig. 3A (**left**) depicts a hypothetical gene X that significantly increases in expression over time in a BCG-group as compared to a placebo group. Fig. 3A (**right**) depicts a two-way interaction where time is treated as a categorical variable, such that the change in expression is evaluated between baseline (day 0) and one other timepoint only (per panel).

Similarly, we applied a linear mixed-effects model with a three-way interaction matrix to determine if the effects of BCG on feature-expression were dependent on scar status (Fig. 3B). For example, Fig. 3B (**left**) depicts a hypothetical gene X that significantly increases in expression over time in the BCG-group as compared to the placebo group in the scar-negative group and not in the scar-positive group. Fig. 3B (**right**) depicts a three-way interaction where time is treated as a categorical variable, such that the change in expression is evaluated between baseline (day 0) and one other timepoint only (per panel). Since skin-biopsies were only collected for a single time-point, we utilized linear models for the gene-expression data from the skin (for each region; epidermis, dermis and hypodermis) with the same two-way and three-way interaction panels. For each comparison, we adjusted for multiple testing using the Benjamini-Hochberg false discovery rate [51] (BH-FDR), applying a BH-FDR cut-off of 10% to assess statistical significance.

### Analyses for secondary objectives

We anticipate that the processes in the skin, captured via spatial transcriptomics, led to measurable changes in blood that, with access to both samples and data sets, will allow us to determine their association. With such association of biomarkers in place, an important study objective is to enable future use of blood-based screening as a proxy for skin biopsies and with that permit scale up in future large clinical trials, aimed to determine how to best deploy BCG for the benefit to the recipients. To this end, we employed methods that integrate multiomics datasets to identify associative patterns across biological layers using unsupervised integrative methods such as MultiOmics Factor Analysis [52] (MOFA), and regularized generalized canonical correlation analysis (RGCCA) [53]. Since baseline gene-expression from skin was not obtained, we only utilized blood and skin-image data from the post-inclusion time-points. To inform the analysis with respect to the baseline levels, we restricted the analysis to significant signatures identified in the analyses addressing the primary objective. This allowed us to identify shared factors across the skin and blood that induce strong effects with respect to individual biological layers also (e.g., skin gene-expression, skin image features, blood cfRNA, blood methylation, and blood proteomics).

The proposed study deployed complex procedures regarding data collection and advanced methods for collecting and processing blood and skin samples on site in Guinea-Bissau. Shipment of the samples under appropriate conditions might pose challenges. However, the study team is experienced in conducting research involving sample collection in this West African setting and have access to the necessary laboratory infrastructure [[54], [55], [56], [57], [58]]. To support correct sample collection, a dermatologist who routinely collects skin samples by punch biopsy was employed to train and supervise the local nurse who collected the biosamples. A secondary aim of the study was thus to evaluate the feasibility of advanced protocols including tissue and blood samples collection in this low resource setting. The results will inform future study design.

### Management of missing data

Given the small sample size and specific inclusion criteria for this proof-of-concept pilot study, a modest set of variables are included in the analytical plan. These variables include randomization group, follow-up day and BCG scar status. Experimental variables include size of inoculum following injection. Sensitivity analyses will address the potential importance of sample processing details (e.g. length of time between sample collection and freezing). With this, missing data is anticipated to be rare and if the missing data might be available in another BHP database, we sought it there. Where data was missing <10% of the time in the final dataset, missing values were imputed. Variables with greater than 10% missing data would have been deemed unsuitable for analysis. In some cases, samples may have quality control failures and need to be removed. To handle such cases, we have specifically included methods such as linear-mixed effects models (for univariate analysis) and MOFA (data integration analysis) that can handle missing data where no data removal is required.

### Publication

The findings of this pilot study will be published in an international peer-reviewed journal. A copy of the findings will be provided to the library of the National Institute of Public Health (INASA) in Guinea-Bissau and submitted to the National Ethics Committee within Health of Guinea-Bissau.

### Protocol status

The study protocol has been approved by the National Committee for Ethics in Health Research of Guinea-Bissau (CNES), reference number 027/CNES/INASA/2023.

## Potential impact

BCG has benefitted billions of people throughout its century of use. Aside from its use against TB, BCG has been tested in several RCTs enrolling adults to test BCG’s effects against COVID-19 during the pandemic. Across these trials, randomization to BCG did not protect against COVID-19 infection but receiving BCG was associated with a reduction in the all-cause mortality risk, when compared to placebo [59]. Yet despite its prolonged use and BCG being the only licensed vaccine against TB, there are many unknowns regarding its immediate and downstream effects within the skin and in the peripheral circulation. Most new TB vaccine trials either include BCG, are built directly upon BCG (modified/recombinant BCG) or are designed as a booster vaccine dose to be administered following initial priming with BCG. When focusing on the specific protection against TB, BCG is thus bound to stay around in the foreseeable future. Both for this reason and due to BCG’s marked non-specific immune training effects, it is crucial to increase our understanding of how the human organism, and its various tissues, respond to BCG.

A major strength of the proposed BCG In Situ experimental study is that we ensured a 50/50 distribution of participants with BCG scars (yes/no), evaluated prior to enrolment by experienced physicians, with adequate power secured to evaluate effects in the different subgroup combinations. This setup enabled us to study effects of BCG in the skin and peripheral circulation among participants that are likely BCG-naïve, and participants that previously received BCG, as documented by the presence of a BCG scar. Crucial differences in the immune response between first vaccination with BCG and revaccination might thus be revealed.

There are many different BCG strains in production worldwide, and they differ markedly in bacterial viability, RNA content and innate immune activation [60]. A strength of the present study is that we used BCG-Danish, which is a widely used vaccine and among the most potent vaccines for which beneficial non-specific effects has been reported in trials from Guinea-Bissau [7,8], Uganda [9] and India [10].

Major limitations include that only women were enrolled in the current study design, and that all are adults. The modest sample size for this initial study also does not allow for integrating a multitude of host variables (e.g., health history, prior pregnancies, nutritional status) on the local response to BCG vaccination. All these variables can however be investigated in larger follow-up studies to be done after feasibility has been documented in this study. The study design includes the collection of only one skin biopsy per study participant. This strategy ensures minimal distress to study participants, while allowing the study team to introduce this new technique in a study setting where such procedures are rare. Yet, the chosen approach will limit the ability to identify robust changes over time within the same individuals. Future studies can consider performing multiple biopsies at one injection site or administering multiple injections that are biopsied separately.

While this pilot study focussed on early responses to BCG vaccination, a pilot study performed in Baboons found important age-dependent differences in skin responses to BCG vaccination eight weeks, but not 25 weeks following intradermal BCG vaccination [61]. Both age of the vaccinee and time since vaccination (or exposure to TB/mycobacteria) may be of importance for the *In Situ* immune response and thus of relevance for future studies applying this protocol to assess differences in response across time and age.

It is a possibility that previous exposure to BCG, TB (including latent TB) and/or environmental mycobacteria, or the maturation of the immune system itself and its barriers (the skin), might inhibit the usefulness of conducting the proposed experiment in adults, or it may reveal why intradermal BCG in adults is less efficacious against *Mtb* than intradermal BCG provided to newborns [62]. Nevertheless, the study is likely to pave the road for similar experiments in the most important cohort that receives BCG – neonates and infants.

With the cellular and molecular events following BCG intradermal administration having never been fully assessed, despite BCG having been administered to billions over many decades via this route, the proposed project delves into questions that have for too long remained unanswered, or only partially answered. The results of the proposed project will thus, irrespective of the effects detected, be of substantial importance to the scientific community.

## Protocol validation

Not applicable.

## Limitations

Not applicable.

## Availability of data and materials

The data collected within the proposed project will be available upon reasonable request sent to both the corresponding author (FSB, fschaltz-buchholzer @ health.sdu.dk) and the senior author (NA, n.akuvy@ gmail.com).

## Funding

This work was supported by unrestricted university operating/start-up funds available to Kollmann T.R.

## CRediT authorship contribution statement

**Frederik Schaltz-Buchholzer:** Conceptualization, Methodology, Validation, Writing – original draft, Resources, Project administration. **Ole Bæk:** Conceptualization, Methodology, Validation, Writing – original draft. **Amrit Singh:** Conceptualization, Methodology, Software, Validation, Visualization, Resources, Writing – original draft. **Elsi Cá:** Writing – review & editing, Resources, Project administration. **Isaquel da Silva:** Writing – review & editing, Resources, Project administration. **Peter Aaby:** Resources, Project administration, Writing – review & editing. **Christine Stabell Benn:** Conceptualization, Methodology, Validation, Writing – review & editing, Resources, Project administration. **Tobias R. Kollmann:** Conceptualization, Methodology, Software, Validation, Writing – review & editing. **Nelly Amenyogbe:** Conceptualization, Methodology, Software, Validation, Visualization, Resources, Writing – original draft.

## Declaration of competing interest

The authors declare that they have no known competing financial interests or personal relationships that could have appeared to influence the work reported in this paper.

## Data Availability

No data was used for the research described in the article.

## Appendix Supplementary materials

Image, application 1

Image, application 2
