# Supplementary material for: The BCG in situ study – novel techniques applied to a 100-year-old vaccine
Source: MethodsX. 2025 Nov 7;15:103712. doi: 10.1016/j.mex.2025.103712 (PMC12681442; doi:10.1016/j.mex.2025.103712)
Supplement: Supplementary file 2 [file mmc2.docx]

**English version:**

<https://vimeo.com/1124453750?ts=0&share=copy> (Password: InSitu_english).

**Portuguese version:**

<https://vimeo.com/760259224?ts=0&share=copy> [Password: bcginsitu2023!]
